# Clinical Validation of the UKMS Register Minimal Dataset utilising Natural Language Processing

**DOI:** 10.23889/ijpds.v1i1.288

**Published:** 2017-04-19

**Authors:** Rod Middleton, Ashley Akbari, Hazel Lockhart-Jones, Jemma Jones, Charlotte Owen, Stella Hughes, Richard Gain, David Ford

**Affiliations:** Swansea University; Shrewsbury and Telford NHS Trust; Belfast NHS Trust; Clinithink

## Objectives

The UK MS Register is a research project that aims to capture real world data about living with Multiple Sclerosis(MS) in the UK. Launched in 2011, identified data sources were: Directly from People with MS (PwMS) via the internet, from NHS treatment centers via `traditional' database capture and by linkage to routine datasets from the SAIL databank.

Data received from the NHS, though `gold standard' in terms of diagnosis, is dependent on clinical staff finding both time and information to enter into a clinical system. System implementations across the NHS are variable, as is clinical time. Therefore, we looked to other complementary methodologies. 

## Approach

The Clix enrich natural language processing (NLP) software was chosen to see if it could capture a portion of the MS Register minimum clinical dataset, the software matches clinical phrases against SNOMED-CT.

40 letters, from 2 NHS Trusts, from 28 patients were loaded. The letters were a mix of MS patients with differing disease subtypes and were dictated by Neurologists, Specialist General Practitioners and MS Specialist Nurses. 20 of the letters were in docx format and 20 as PDF.

The letters were parsed by a domain expert for clinical content, scored by data item for sensitivity and specificity. Next the output from the software was scored by another researcher to see if the 12 relevant clinical concepts from the Register dataset had been elicited. Lastly a ruleset was created to look for particular clinical concepts and scored in the same way.

## Results

Of the 40 letters one failed to load, the rest were analysed for the specific data items. Date related items were clearly challenging, with only 7% of appointment dates being matched and 22% for date of diagnosis.

MS Type (93.3%) and EDSS score (93.75%) were well recognised, additionally symptoms of MS that would be poorly reported in traditional databases were recognised, with fatigue being well highlighted (78.5%) and gait and walking issues (68.7%)

Of concern, were a number of false positive results in DMT’s with 15% patients being identified as being on a DMT when this was just being `considered'.

## Conclusion

The NLP pathway could be extremely useful for obtaining hard to capture clinical data for the Register. Further work is needed to reduce errors, even with the current minimal configuration, it's possible to ascertain MS Type, functional score of MS, current medication and potentially disabling symptomology within the condition.

